# Physico-Chemical Changes Induced by Gamma Irradiation on Some Structural Protein Extracts

**DOI:** 10.3390/biom13050774

**Published:** 2023-04-29

**Authors:** Maria Stanca, Carmen Gaidau, Traian Zaharescu, George-Alin Balan, Iulia Matei, Aurica Precupas, Anca Ruxandra Leonties, Gabriela Ionita

**Affiliations:** 1 Leather Research Department, Research and Development National Institute for Textiles and Leather-Division Leather and Footwear Research Institute, 93, Ion Minulescu Street, 031215 Bucharest, Romania; 2 INCDIE ICPE CA, 313 Splaiul Unirii, 030138 Bucharest, Romania; 3 “Ilie Murgulescu” Institute of Physical Chemistry of the Romanian Academy, 202 Splaiul Independentei, 060021 Bucharest, Romania

**Keywords:** keratin, collagen, bovine gelatin, fish gelatin, riboflavin, μDSC, circular dichroism spectroscopy, EPR spectroscopy, IR spectroscopy

## Abstract

In this study, the effect of gamma irradiation (10 kGy) on proteins extracted from animal hide, scales, and wool was evidenced by calorimetric (μDSC) and spectroscopic (IR, circular dichroism, and EPR) methods. Keratin was obtained from sheep wool, collagen and bovine gelatin from bovine hide, and fish gelatin from fish scales. The μDSC experiments evidenced that gamma irradiation influences the thermal stability of these proteins differently. The thermal stability of keratin decreases, while a resistance to thermal denaturation was noticed for collagen and gelatins after gamma irradiation. The analysis of the IR spectra demonstrated that gamma irradiation determines changes in the vibrational modes of the amide groups that are associated with protein denaturation, most meaningfully in the case of keratin. As evidenced by circular dichroism for all proteins considered, exposure to gamma radiation produces changes in the secondary structure that are more significant than those produced by UV irradiation. Riboflavin has different effects on the secondary structure of the investigated proteins, a stabilizing effect for keratin and fish gelatin and a destabilizing effect for bovine gelatin, observed in both irradiated and non-irradiated samples. The EPR spectroscopy evidences the presence, in the gamma-irradiated samples, of free radicals centered on oxygen, and the increase in their EPR signals over time due to the presence of riboflavin.

## 1. Introduction

Gamma irradiation at low to medium doses of 5–10 kGy is a known microbial control method and safety measure in food and cosmetic industries [1], as well as a method largely used in medical treatments [2], in particular for cancer therapy. There are studies that indicate this procedure increases the production of extracellular matrix (ECM) that contains large amounts of collagen. The presence of collagen confers resistance to radiation, which has an effect on the reduction in cell mortality in some tumors [3]. On the other hand, gamma irradiation causes the fragmentation of the α chains of collagen in tissues such as bones, determining the loss of connectivity in collagen networks and, by consequence, bone fractures [2]. Gamma irradiation also determines the radiolysis of water, which further generates free radicals. These radicals, by interaction with the components of the ECM, including proteins such as collagen, gelatin and keratin, can initiate a series of chemical processes such as deamination, decarboxylation, reduction in the number of disulfide bonds, oxidation of sulfhydryl groups, and hydrolysis of peptide bonds [4].

Nevertheless, the gamma sterilization method has some advantages that lend it to use for large-scale applications, including the low cost, the small increase in temperature of the irradiated materials, and no production of toxic residues [5]. The negative impact on a system or material can be limited by controlling the dose of gamma radiation, or by introducing other compounds that can inhibit the action of free radicals in the system.

In the case of proteins extracted from the ECM, the extraction procedure itself can modify the native structure of these proteins. In addition, the extracts can be modified by grafting other molecules, depending on their envisaged applications. Thus, gamma-irradiated collagen can find application in tissue engineering [6], or can be used as a component of interpenetrated networks along other biocompatible polymers to find application in wound dressing [7]. In fact, combining polysaccharides with proteins from the ECM represents a common procedure to obtain new medical devices [8]. Recently, the characteristics of a collagen/chitosan membrane obtained by the casting method, and the effect of lemon grass oil as an antimicrobial agent, were reported [9]. In similar studies, keratin hydrolysates were physico-chemically characterized and tested for cellular proliferation [10].

In this study, we aimed to find evidence of structural changes induced by gamma irradiation or UVA irradiation in the presence of riboflavin in different samples of known protein constituents of the ECM extracted from bovine hide, fish scales, and sheep wool. The physico-chemical methods involved were differential scanning microcalorimetry (μDSC), IR spectroscopy, circular dichroism (CD), and electron paramagnetic resonance (EPR) spectroscopy. Riboflavin was used in this investigation, considering our previous study regarding its role in generating collagen crosslinking [11]. This process has already found application in the treatment of eye diseases, such as keratoconus and keratitis of different etiologies, as well as vein or dentine diseases [12,13,14].

## 2. Materials and Methods

### 2.1. Materials

Spin traps 5,5-dimethyl-1-pyrroline-N-oxide (DMPO) and N-tert-butyl-α-phenylnitrone (PBN) were purchased from Aldrich (St. Louis, MO, USA). Riboflavin was purchased from Carl Roth (Karlsruhe, Germany). 

### 2.2. Extraction Procedures

The extraction procedures utilized to obtain the proteins used in this study were as follows.

Keratin hydrolysate was obtained by alkaline–enzymatic hydrolysis in two steps. In the first step, the alkaline hydrolysate was obtained at 80 °C by a previously described method [10]. Briefly, sheep wool was washed and degreased using NH_4_OH 4% *w*/*w*, Na_2_CO_3_ 1% *w*/*w*, and Boron SE 0.6% *w*/*w* for 2 h at 40 °C, then washed up to neutral pH and cut into small pieces using a grinding machine (La Minerva, Minerva Omega, Bologna, Italy). Wool was mixed with NaOH 2.5% *w*/*w* solution in a stainless steel vessel equipped with automatic temperature control and mechanically stirred for 4 h at 80 °C. The obtained hydrolysate was used in the second step for the enzymatic hydrolysis using 1% w/w of the enzyme Valkerase (BioResource International, Durham, NC, USA). The keratin hydrolysate was centrifuged for 15 min at 6000 rpm (Eppendorf 5804, Wien, Austria) and then lyophilized by freeze drying using a DELTA 2-24 LSC freeze dryer (Osterode am Harz, Germany).

Bovine collagen was obtained from bovine delimed hide. Bovine hide was cut into small pieces and washed several times with deionized water. The collagen was extracted at neutral pH in a water bath at 90 °C for 6 h. The obtained product was filtered and dried in an oven at 60 °C.

Bovine gelatin was obtained by acid hydrolysis of bovine delimed hide mixed with a certain amount of distilled water, heated at 90 °C for 5 h. The pH was set at 5.5 using a solution of 1 M acetic acid, and was controlled and adjusted every hour. The obtained gelatin was filtered and dried in an oven at 60 °C.

Fish gelatin was obtained from fish scales following a method described by Akagunduz et al. [15]. Briefly, shredded fish scales were washed with NaCl 5% *w*/*v* and NaOH 4% *w*/*v* to remove non-collagen proteins. After the alkali treatment, scales were treated with n-butanol (10 mL/100 mL) three times for 30 min to remove fat. Scales were washed with distilled water between steps. In order to partially remove the mineral content, scales were treated for 16 h with a solution containing EDTA and, thereafter, with acetic acid 99% for 3 h. The extraction was made in distilled water at 60 °C for 12 h. The obtained gelatin was filtered and dried in an oven at 60 °C.

### 2.3. Instrumentation and Sample Preparation

Solutions of bovine collagen, bovine gelatin, and fish gelatin were prepared by dissolving the protein to a 0.25 mg/mL concentration in 0.18 M or 15.7 M acetic acid, in the absence or presence of riboflavin 0.01% (0.1 mg/mL). Keratin was dissolved in water to obtain a 0.46 mg/mL solution.

The physico-chemical characterization through IR, circular dichroism, and EPR spectroscopies, as well as μDSC, was performed for samples prior to and after irradiation with UVA light or gamma radiation. A dose of 10 kGy over an irradiation time of 5 h, in air, was used. 

#### 2.3.1. Infrared Spectroscopy

The IR spectra of dried samples obtained by the evaporation of protein solutions prior to and after exposure to radiation were recorded on a Nicolet i-S10 FTIR spectrophotometer (Thermo Scientific, Waltham, MA, USA). For secondary structure estimation, the FTIR spectra in the amide I (1600–1700 cm^−1^) spectral region were deconvoluted to the minimum number of Gaussian components. The negative maxima in the fourth derivative of the FTIR spectra were used as deconvolution input parameters, as indicated in the literature [16,17]. Band positions and widths were constrained within reasonable limits. Correlation coefficients and standard errors were >0.99 and <0.002, respectively. In order to assess the relative contribution of each secondary structure component, the area of the corresponding band was divided into the sum of areas of all the components considered.

#### 2.3.2. Circular Dichroism (CD) Spectroscopy

The CD spectra of protein solutions were recorded on a J-815 spectrometer (JASCO International Co., Ltd., Tokyo, Japan) equipped with a Peltier-type temperature controller. Measurements were conducted at three temperatures in the range of 298–308 K, which is the range of the endothermic signal obtained by μDSC. The CD spectra were recorded using a 0.5 mm path length cuvette, between 190 nm and 260 nm, with standard sensitivity and at a scan rate of 50 nm/min. The other parameters were set as follows: band width 1 nm, response 4 s, and data pitch 1 nm. Each CD spectrum represents the average of three individual scans. The CD spectrum of the baseline (water or acetic acid 0.18 M) was subtracted. The effect of gamma irradiation on the secondary structure was investigated for the protein samples containing riboflavin. After gamma irradiation, no CD signal was detected for riboflavin; therefore, the CD spectrum of riboflavin was only subtracted for the samples recorded prior to irradiation.

#### 2.3.3. Differential Scanning Microcalorimetry (μDSC)

The thermal stability of protein solutions was measured using the μDSC7 evo calorimeter (Setaram, Caluire, France). Two Hastelloy cells, the sample cell containing 100 μL of sample and the reference cell filled with the same volume of solvent, were heated from 278 K to 363 K using a scan rate of 1 K min^−1^. Data collection and processing were performed with the Calisto v.1077 software supplied by the instrument manufacturer, using a linear baseline. The temperature of denaturation (T_peak_) and the denaturation enthalpy change (ΔH) of the samples were determined. The influence of gamma irradiation on the samples containing riboflavin was studied.

#### 2.3.4. Electron Paramagnetic Resonance (EPR) Spectroscopy

The EPR spectra of spin adducts were recorded on a JEOL FA 100 spectrometer (Tokyo, Japan) equipped with a cylindrical-type resonator TE011, with a frequency modulation of 100 kHz, microwave power of 0.998 mW, sweep time of 60 s, modulation amplitude of 1 G, time constant of 0.1 s, modulation width of 1 G, and a magnetic field scan range of 100 G. The hyperfine coupling constants of the spin adducts were obtained from simulation of the experimental spectra using the Winsim software [18,19]. 

#### 2.3.5. UVA and Gamma Irradiation

UVA light exposure of protein samples was performed with a mercury arc lamp (500 W, LOT-Quantum Design, Darmstadt, Germany) at 370 nm for 30 min. The gamma irradiation process was performed with an irradiator M-38 GAMMATOR (Isomedix, Parsippany, NJ, USA) using a ^137^Cs source, in air, at room temperature, at a dose rate of 2 kGy/h.

## 3. Results and Discussions

### 3.1. IR Spectroscopy

**Figure 1:**
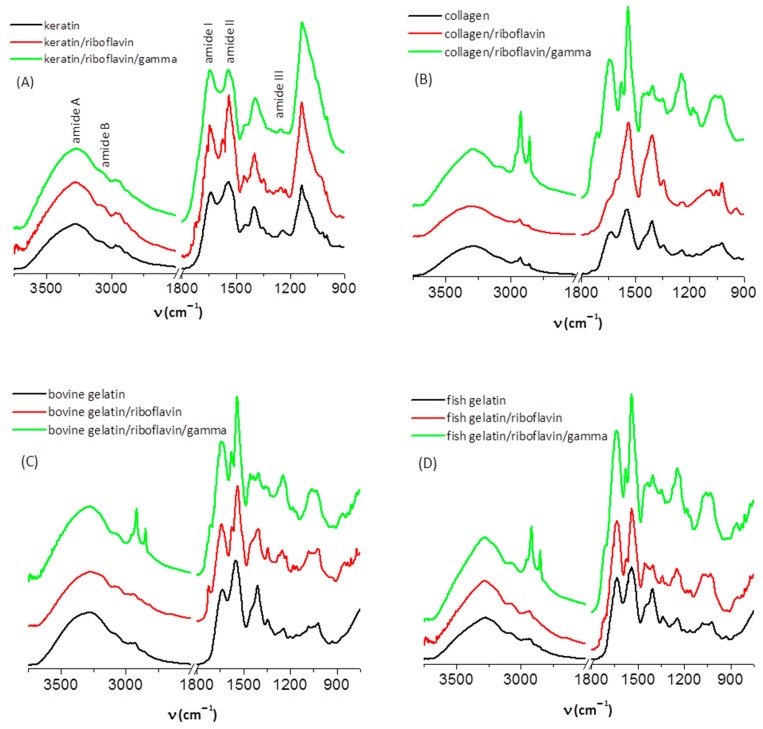
The IR spectra of (**A**) dried keratin, (**B)** collagen, (**C**) bovine gelatin and (**D**) fish gelatin samples.

The investigation of the protein samples by IR spectroscopy was performed in order to identify the characteristic signals of the main functional groups by referring to the literature data [20,21,22,23] (see Figure 1, Table 1 and Tables S1–S3 in the Supplementary Materials file), and to estimate the secondary structural content of the protein samples, prior to and after irradiation. We aimed to assess the extent of conformational changes that are induced in the protein structure by gamma irradiation with a dose of 10 kGy. This information can be retrieved by correlating the positions and intensities of characteristic amide vibrational modes to specific secondary structure elements of the polypeptide chain (α-helix, β-sheet, β-turn, random coil).

First, we discuss the results regarding keratin. The amide A band of the three keratin samples under investigation (keratin, keratin/riboflavin, and keratin/riboflavin/gamma-irradiated) has a maximum at 3275 cm^−1^. It arises from N-H and O-H stretching vibrations of the polypeptide chain, and its broad feature was previously considered an indication for the presence of twisted β-sheet structures formed via hydrogen bonding [24]. 

The amide I spectral region (1600–1700 cm^−1^) comprises C=O stretching (~80%), N-H bending, and C-N stretching (20%) vibrations of the polypeptide chain that are correlated to the backbone conformation [21]. This signal is intense and can be used to obtain an estimate of the secondary structural content of the protein. The main amide I peak is observed at 1639 cm^−1^ and 1642 cm^−1^ for the keratin and keratin/riboflavin samples, and at 1644 cm^−1^ for the keratin/riboflavin/gamma-irradiated sample. 

The amide II spectral region (1500–1600 cm^−1^) contains contributions from N-H in-plane bending and C-N stretching vibrations (1540–1560 cm^−1^), as well as from C-N and C-C stretching (1520–1540 cm^−1^). It is less sensitive to conformational changes as compared to the amide I vibrational mode, and is mostly indicative of the protonation state of the peptide unit [25]. 

The amide III band (1220–1330 cm^−1^) is associated with C-N stretching, N-H bending (~30% each), C-C stretching (~20%), and C-H bending (~10%) vibrations [21]. Despite having low intensity, the amide III mode is sensitive to the nature of the side chains and to hydrogen bonding [25,26], and it can sometimes be used, complementary to the amide I mode, for conformational analysis.

As stated above, the amide I band (1639 cm^−1^) of keratin slightly shifts to larger wavenumbers in the presence of riboflavin (1642 cm^−1^), and more so after exposure to gamma radiation (1644 cm^−1^). When the IR spectra were normalized (using the 1399 cm^−1^ C-H bending band as a reference) in order to better observe any differences in band intensity, position, or shape, we noted a decrease in the intensity of the amide A band after irradiation. This may indicate some degree of degradation of the amide group (primary structure alteration) [9,27]. The most poignant spectral change caused by irradiation is the change in the intensity ratio of the amide I and amide II bands from 0.82 in the keratin/riboflavin sample to 1 in the keratin/riboflavin/irradiated sample. This change evidences the disruption of the intrinsic hydrogen bonding structure of the protein due to conformational changes (secondary structure alteration). An increase in the amide I/amide II intensity ratio was shown to correlate to an increase in the contribution of ordered structures [9]. In our case, the opposite trend is observed; therefore, we expect irradiation to cause an increase in the random coil contribution at the expense of β and α ordered structures. This can be confirmed by a quantitative assessment of the secondary structure. 

**Figure 2:**
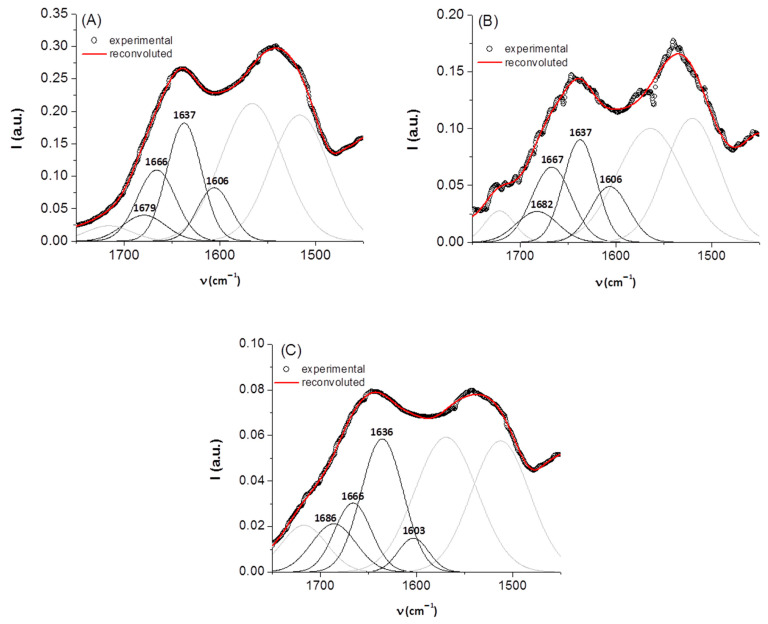
Deconvolution of the amide I region of the IR spectra of keratin samples: (**A**) keratin, (**B**) keratin/riboflavin, (**C**) keratin/riboflavin/gamma-irradiated; for clarity, band components not belonging to the region of interest are depicted in grey.

For secondary structure estimation, we focused on the amide I region. In this spectral domain, the secondary structure components of a protein absorb at different positions, as follows: β-sheet (1610–1635 cm^−1^), random coil (1635–1645 cm^−1^), α-helix (1645–1665 cm^−1^), β-turn (1662–1682 cm^−1^), and β-sheet antiparallel (1682–1689 cm^−1^) [24,28,29]. It is known that some spectral features appearing as faint in the IR spectrum can become more prominent in the second [30] or fourth [16,17] derivative of the spectrum. For this reason, in order to have a more precise initial estimate of the component bands (in terms of number and position), the negative maxima in the fourth derivative of the IR spectrum of keratin were used. As such, the amide I band was fit with four Gaussian components (initially set at 1607, 1635, 1653, and 1685 cm^−1^). The bands resolved after deconvolution are presented in Figure 2 and their relative contributions are listed in Table 2, together with their assignment. We note that the presence of riboflavin and the exposure to gamma radiation do not determine significant shifts in the position of these bands, but change their relative contributions substantially. The effect of irradiation is the increase in unordered random coil structures at the expense of β-sheet ordered structures.

In addition to the vibrational modes observed for keratin, the collagen (Figure 1B), bovine gelatin (Figure 1C), and fish gelatin (Figure 1D) samples exhibited bands in the spectral region of 1000–1100 cm^−1^ that have been interpreted considering the glycation of collagen, and thus ascribed to C-O stretching vibrations of carbohydrates [31]. The main spectral changes induced by gamma irradiation of these samples occur in the spectral regions attributed to C-H stretching and C-H bending vibrations. Thus, irradiation produces a strong increase in the intensity of the C-H asymmetric and symmetric stretching bands, accompanied by a strong decrease in the C-H bending mode at ~1405 cm^−1^. For collagen and bovine gelatins, a shift of the amide II band from ~1547 cm^−1^ in the native samples to ~1540 cm^−1^ in the samples containing riboflavin and in the irradiated samples is also observed. Some authors explain such a shift to lower wavenumbers by the fibrillogenesis of collagen [31].

For deconvoluting the spectra of collagen and gelatins, four Gaussian components were initially set at 1615, 1638, 1651, and 1685 cm^−1^, as predicted by the fourth derivative of the IR spectra. The bands resolved after deconvolution are presented in Figures S1–S3, and their relative contributions are listed in Tables S4–S6. The secondary structural content of bovine gelatin was estimated at 13% β-sheet, 48% random coil, 26% α-helix, and 13% β-turn. No significant changes in these values were observed upon interaction with riboflavin or after gamma irradiation (Table S5). For fish gelatin, the predicted secondary structural content of 16% β-sheet, 47% random coil, 25% α-helix, and 12% β-turn was not altered significantly upon interaction with riboflavin or by gamma irradiation (Table S6). The secondary structural contents of both bovine and fish gelatins evidence a higher degree of denaturation as compared to bovine collagen.

### 3.2. Circular Dichroism (CD) Spectroscopy

The secondary structures of the protein samples were further analyzed using CD spectroscopy. Below, the changes induced by temperature, the presence of riboflavin, and irradiation with UV or gamma radiation are described.

**Figure 3:**
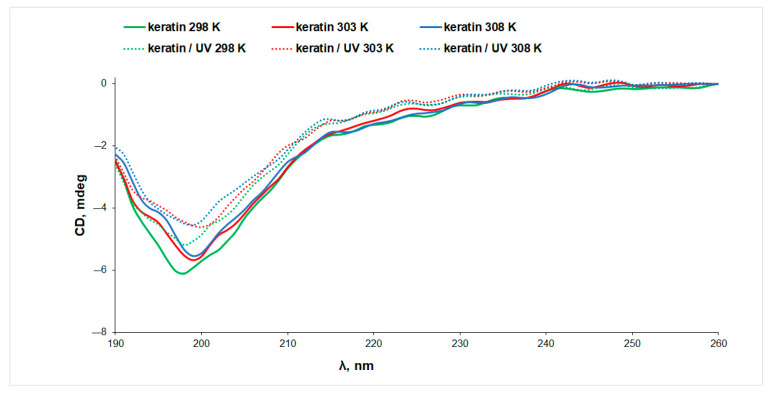
The CD spectra of keratin (line) and UV-irradiated keratin (dotted line) aqueous solutions measured at different temperatures.

The CD spectra of aqueous keratin solutions in the absence and presence of riboflavin, prior to and after exposure to UV radiation, at three temperatures, are presented in Figure 3. It is known from the literature [32] that the α-helical CD spectrum of α-keratin shows two negative minima at 222 nm and 208 nm, and a positive maximum at 190 nm. The CD spectra presented in Figure 3 display a single broad negative peak at ~200 nm, indicating a low content of α-helix conformation in the case of our keratin sample. This observation is in agreement with the helical content of only ~28% estimated from IR data.

**Figure 4:**
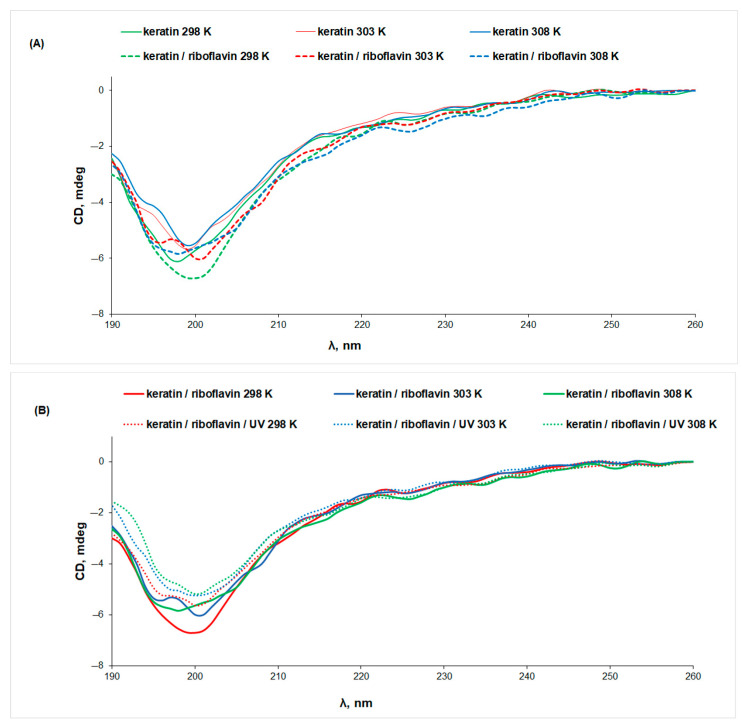
The CD spectra of (**A**) keratin (line) and keratin/riboflavin (dotted line), (**B**) keratin/riboflavin (line) and keratin/riboflavin/UV-irradiated (dotted line) aqueous solutions measured at different temperatures.

Both the increase in temperature and the exposure to UV radiation determine a decrease in the CD signal of keratin, pointing to changes in the protein secondary structure associated with protein denaturation. More intense CD signals were obtained for keratin samples in the presence of riboflavin, indicating a stabilizing effect of riboflavin on the protein structure as a result of interaction (Figure 4A). This observation can be correlated to the slight increase in the content of ordered structures at the expense of the random coils, which decrease from 42% in keratin to 35% in keratin/riboflavin (Table 1).

**Figure 5:**
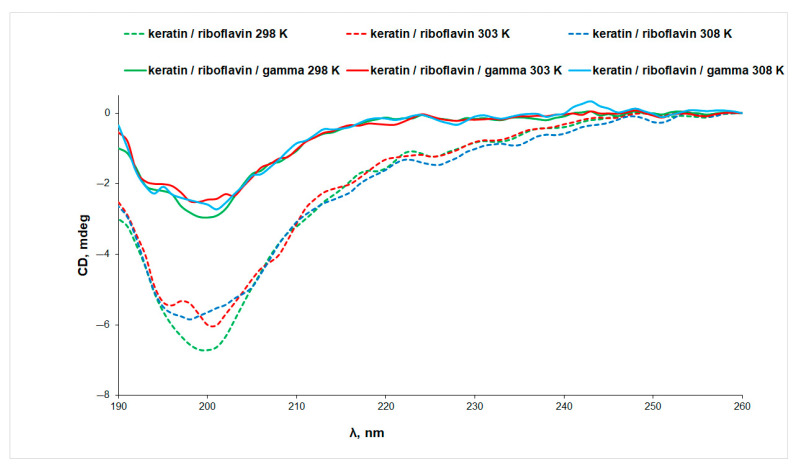
The CD spectra of keratin/riboflavin (line) and keratin/riboflavin/gamma-irradiated (dotted line) aqueous solutions measured at different temperatures.

UV irradiation determines a 13% decrease in the intensity of the CD signal of the keratin/riboflavin sample, pointing to protein denaturation (Figure 4B). Gamma irradiation of the keratin/riboflavin aqueous solution has an even larger denaturation effect, manifesting in an approximately 50% decrease in the ellipticity value at all temperatures (Figure 5). This correlates to the increase in random coil content at the expense of the β-sheet and α-helix conformations that was observed by IR spectroscopy.

**Figure 6:**
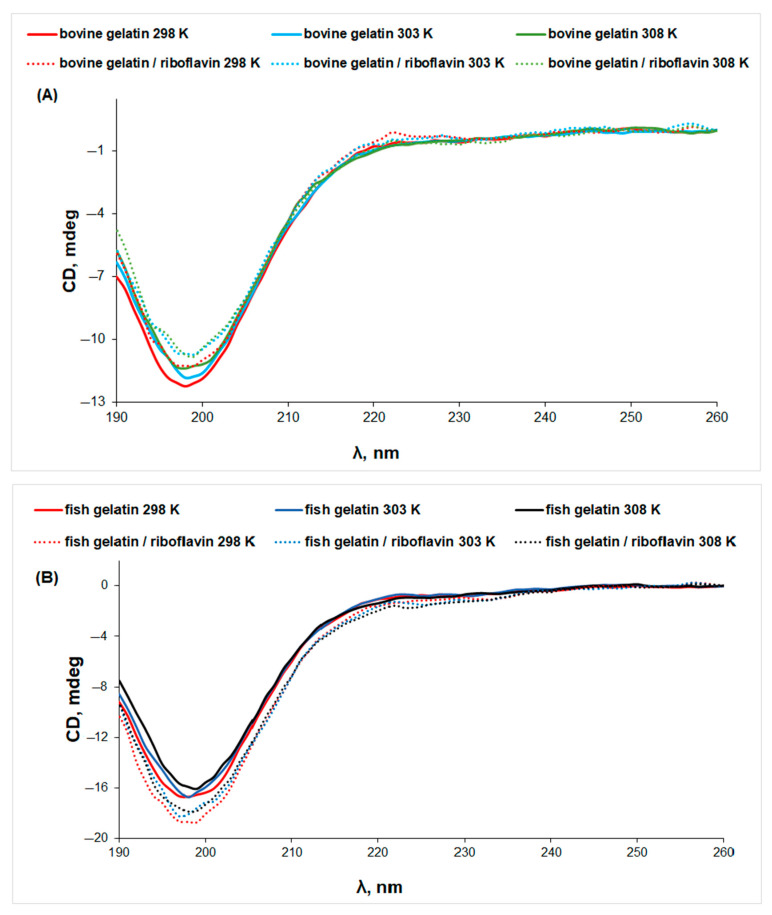
The CD spectra of gelatin (line) and gelatin/riboflavin (dotted line) solutions in acetic acid 0.18 M at different temperatures: (**A**) bovine gelatin and (**B**) fish gelatin.

The CD spectrum of native collagen presents a positive maximum peak at 220 nm and a negative minimum peak at around 198 nm, characteristic for the triple-helix conformation [33]. Gelatin is a protein obtained by breaking the triple-helix structure of collagen [34]. It is known that the positive peak at 220–230 nm characteristic to the triple-helix conformation disappears completely as a result of collagen denaturation [35,36]. For bovine and fish gelatin solutions, the CD spectra (Figure 6) present only a minimum peak at ~199 nm, which can be assigned to the unordered structure of the protein, while the maximum at 222 nm is not observed, which points to denatured collagen structures [37], in accordance with the IR data. The temperature increase determines conformational changes associated with the denaturation of both gelatins. 

In the presence of riboflavin, the CD signal presents different trends: the negative ellipticity decreases for bovine gelatin, suggesting denaturation, while the CD signal increases for fish gelatin. Thus, riboflavin presents a two-fold action: (i) it induces a stabilizing effect on the fish gelatin structure and (ii) increases the denaturation of bovine gelatin.

**Figure 7:**
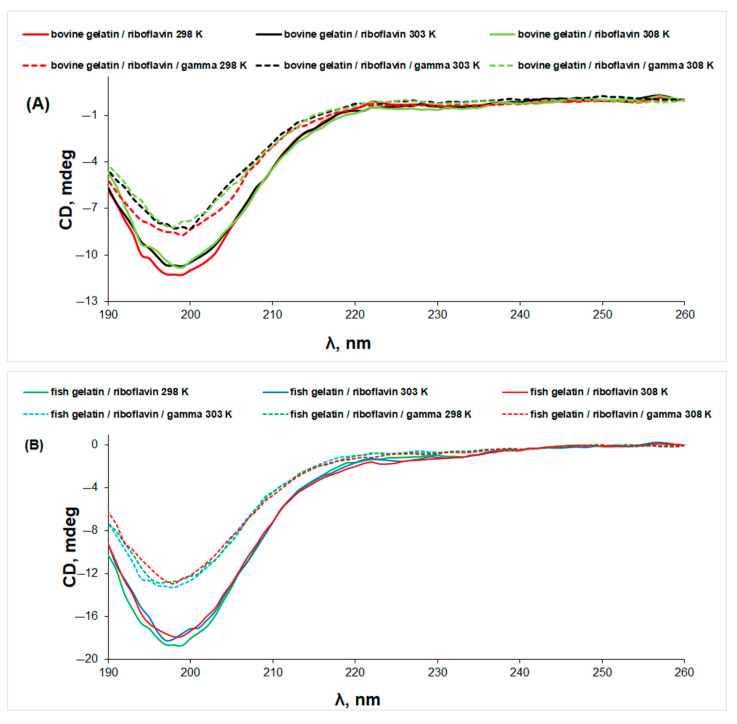
The CD spectra of gelatin/riboflavin (line) and gelatin/riboflavin/gamma-irradiated (dotted line) solutions in acetic acid 0.18 M at different temperatures: (**A**) bovine gelatin and (**B**) fish gelatin.

Exposure to gamma radiation generates a further denaturation of gelatin/riboflavin samples, without changing the shape of the CD spectra (Figure 7). The same effect is observed for the collagen/riboflavin solution (Figure S4).

### 3.3. Differential Scanning Microcalorimetry (μDSC)

The thermal stability of keratin, gelatin, and collagen solutions was investigated using μDSC, and the parameters of denaturation (peak temperature and denaturation enthalpy change) are presented in Table 3. The μDSC signal of all samples presented a broad endothermic peak attributed to the helix to random coil state transition [36,38,39]. 

**Figure 8:**
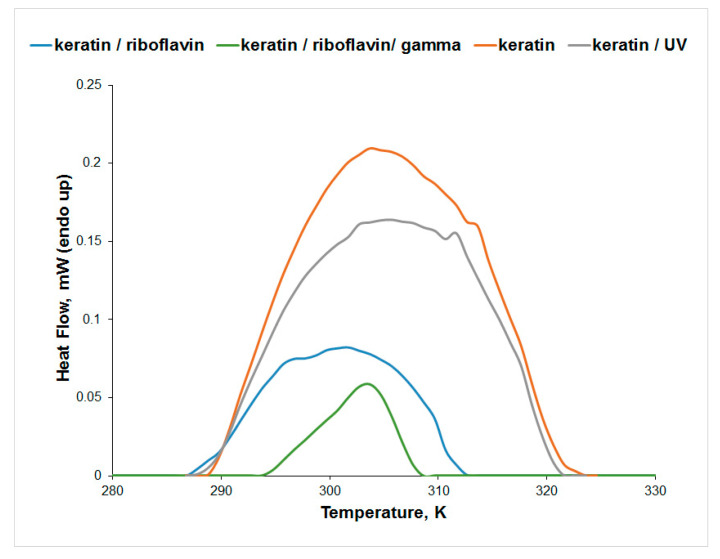
Effect of gamma and UV irradiation on keratin thermal denaturation in water.

The μDSC measurements for the aqueous solution of keratin (Figure 8) indicate a higher thermal stability of keratin after UV irradiation, while the denaturation enthalpy change slightly decreases as a result of the conformational changes observed in the CD spectra. The presence of riboflavin decreases the thermal stability of keratin in water, while gamma irradiation increases the thermal stability of the keratin/riboflavin sample. Although the T_peak_ value increases for the keratin/riboflavin gamma-irradiated sample, the denaturation enthalpy change is significantly reduced. This is a result of the keratin unfolding observed in the CD spectra, which causes the exposure of the hydrophobic domains to the aqueous solution.

A conclusion on the influence of the presence and concentration of acetic acid on the thermal denaturation of keratin can be drawn by comparing the data in Table 3 and Table S7. When keratin was dissolved in acetic acid 15.7 M, significant decreases in the T_peak_ and denaturation enthalpy change values were observed, indicating a destabilization effect, induced by the high concentration of co-solvent. Gamma irradiation of the keratin/riboflavin sample prepared in concentrated acetic acid solution did not change the value of T_peak_, but the denaturation enthalpy change slightly decreased, revealing a somewhat higher exposure of hydrophobic regions of protein to the solvent.

**Figure 9:**
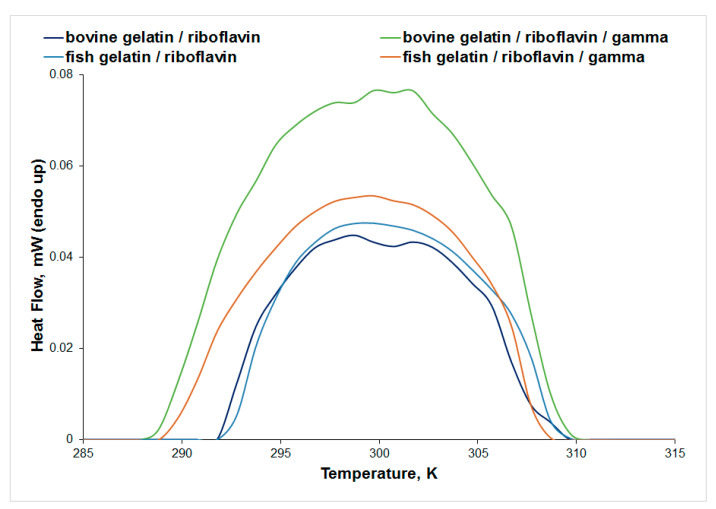
The μDSC signal for the thermal denaturation of bovine and fish gelatin solutions in acetic acid 0.18 M.

The influence of gamma irradiation on the thermal stability of gelatin/riboflavin samples in acetic acid 0.18 M is presented in Figure 9. The endothermic peak observed for gelatin corresponds to the helix-to-coil transition, as a result of the dissociation of the triple helices upon heating [40]. An increase in the thermal stability of the bovine gelatin/riboflavin sample was observed after irradiation, while no significant change was recorded in the μDSC profile of the fish gelatin/riboflavin sample. These results are in agreement with the IR data that showed no spectral changes for the fish gelatin/riboflavin sample as a result of irradiation.

Gamma irradiation of bovine gelatin in acetic acid 15.7 M in the presence of riboflavin decreases the thermal stability of the protein (T_peak_ decreases from 302.64 K to 301.45 K, see Table S7), while the same system in acetic acid 0.18 M is more stable to heat after gamma irradiation (T_peak_ increases from 298.96 K to 301.32 K, Table 3). An important change in thermal stability was observed mainly for fish gelatin: when using a higher concentration of acetic acid, the value of T_peak_ increases from 299.49 K to 303.14 K, pointing to a stabilizing effect. An important decrease in the denaturation enthalpy change value was obtained for the fish gelatin/riboflavin sample in acetic acid 15.7 M after gamma irradiation. Gamma irradiation induces a slight increase in the thermal stability of the collagen/riboflavin sample (Figure S5).

### 3.4. EPR Spectroscopy

Two spin trapping agents, DMPO and PBN, have been used in this study to evidence free radical species that can form in protein/riboflavin systems after gamma irradiation. No EPR signal was recorded for the irradiated solutions in the absence of spin trapping agents. The spin trap was added to the protein solution containing riboflavin immediately after irradiation. The samples were then immersed in liquid nitrogen and the EPR spectra were recorded after 1 h, and again after 2 h. It is worthy of note that the samples were kept on the lab bench between the two measurements, and thus exposed to daylight.

**Figure 10:**
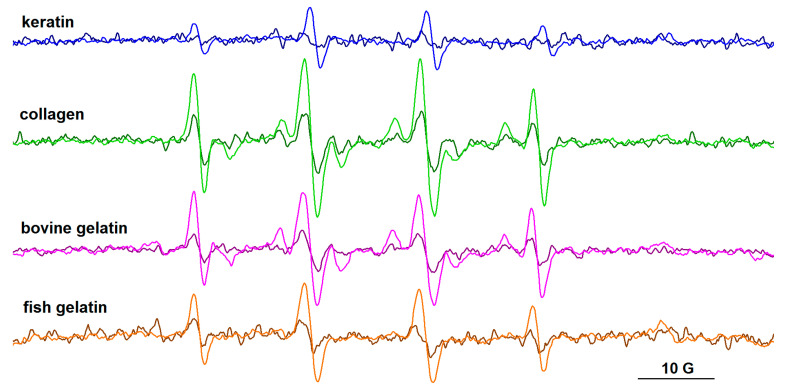
The EPR spectra of DMPO adducts formed after gamma irradiation (10 kGy) in solutions of keratin, collagen, bovine gelatin, and fish gelatin, in the presence of riboflavin. Spectra recorded after 1 h (dark colors) and after 2 h (light colors) from irradiation.

**Figure 11:**
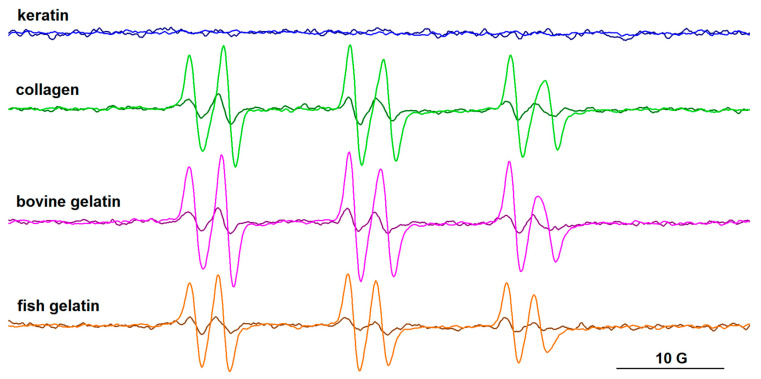
The EPR spectra of PBN adducts formed after gamma irradiation (10 kGy) in solutions of keratin, collagen, bovine gelatin, and fish gelatin, in the presence of riboflavin. Spectra recorded after 1 h (dark colors) and after 2 h (light colors) from irradiation.

The EPR spectra of the spin adducts of DMPO and PBN are presented in Figure 10 and Figure 11, respectively, and their parameters are provided in Table S8. For all protein samples, it can be observed that the intensity of the EPR signal increases over time, and this is due to the presence of riboflavin that generates HO^•^ radicals. The parameters of the ^•^DMPO-OH adducts are similar, with hyperfine coupling constants a_N_~14.8 G and a_H_~13.9 G. 

The PBN adducts evidence six-line signals, their parameters after one hour from irradiation being a_N_ ~14.6 G and a_H_ ~2.6 G. The literature data on carbon-centered radicals formed in aqueous media [41] and in protein samples after gamma irradiation [42] report a_N_ values > 15.5–16 G and a_H_ values > 3 aN for PBN adducts. For PBN adducts formed with oxygen-centered radicals, the literature data indicate lower a_N_ (<15 G) and a_H_ (<3 G) values [41]. These latter values are closer to those obtained for our systems, which indicates, rather than the presence of carbon-centered radicals, the presence of free radicals centered on oxygen. Nevertheless, the slight increase in time of the a_N_ and a_H_ values of the PBN adducts (a_N_~14.8 G, a_H_~2.9 G after two hours from irradiation) may be an indication of the formation of carbon-centered radicals. They could arise from successive reactions of the HO^•^ radicals with the protein chains or with the acetic acid present in solution. As in the case of the DMPO adducts, the intensity of the signal of PBN adducts increases over time. In the case of the keratin sample, radical formation was not observed using PBN as the spin trap. This can also be correlated with the lower intensity signal of the ^•^DMPO-OH adduct evidenced for the keratin sample. 

Exposure of the protein/riboflavin samples to a higher dose of gamma radiation (20 kGy) determined the decomposition of riboflavin in the samples containing collagen or gelatin. The presence of ^•^DMPO-OH spin adducts was only observed for the keratin sample.

## 4. Conclusions

To summarize, the analysis of changes in protein secondary structures induced by gamma irradiation and the presence of riboflavin revealed the influence of the protein source. The μDSC, IR spectroscopy, and CD spectroscopy evidenced that gamma irradiation and riboflavin influence the secondary structure of the ECM-type protein extracts. Riboflavin facilitates the release of free radicals that further interact with the protein chains. However, the exposure to gamma radiation influences the stability and integrity of the molecules. The proteins with higher degrees of ordered structures are more stable against gamma radiation and temperature. Due to their hydrophilic character, collagen and gelatin, when combined with riboflavin, present more stable secondary structures when exposed to gamma radiation as compared to keratin, which has a hydrophobic character. Further studies may involve similar compounds with riboflavin or investigating the effects of antioxidant compounds on the protein structures exposed to gamma irradiation.

## Figures and Tables

**Table 1:** Assignment of the main IR bands of keratin.

Sample	Wavenumber (cm^−1^)	Assignment
keratin	3275, 3060 2963, 2935 1639 (s) 1544 (s) 1448 (w), 1399 1350 (w), 1242 (w) 1136 (s)	O-H, N-H stretching (amide A and B) C-H stretching (asymmetric, symmetric) C=O stretching/N-H bending (amide I) C-N stretching/N-H bending (amide II) C-H bending C-N stretching/N-H bending (amide III) S-O stretching, symmetric (-SO_2_-S-, cystine dioxide)
keratin/riboflavin	3275, 3072 2963, 2935 1642 (s) 1540 (s) 1455 (w), 1398 1347 (w), 1250 1135 (s)	N-H stretching (amide A and B) C-H stretching (asymmetric, symmetric) C=O stretching/N-H bending (amide I) C-N stretching/N-H bending (amide II) C-H bending C-N stretching/N-H bending (amide III) S-O stretching, symmetric (-SO_2_-S-, cystine dioxide)
keratin/riboflavin/gamma	3275, 3068 2971, 2934 1644 (s) 1542 (s) 1394 1251 1133 (s)	N-H stretching (amide A and B) C-H stretching (asymmetric, symmetric) C=O stretching/N-H bending (amide I) C-N stretching/N-H bending (amide II) C-H bending C-N stretching/N-H bending (amide III) S-O stretching, symmetric (-SO_2_-S-, cystine dioxide)

Abbreviations: s—strong, w—weak.

**Table 2:** Secondary structural content of keratin samples.

Sample	Band Assignment	ν (cm^−1^)	% Area
keratin	β-sheet random coil α-helix β-turn	1606 1637 1666 1679	19 42 28 11
keratin/riboflavin	β-sheet random coil α-helix β-turn	1606 1637 1667 1682	22 35 30 13
keratin/riboflavin/gamma	β-sheet random coil α-helix β-turn	1603 1636 1666 1686	9 49 23 19

**Table 3:** Peak temperature (T_peak_) and denaturation enthalpy change (ΔH) obtained from μDSC measurements.

Sample	T_peak_ (K)	ΔH (J/g)
keratin	303.87	2.69
keratin/UV-irradiated	305.50	2.15
keratin/riboflavin	301.68	0.76
keratin/riboflavin/gamma-irradiated	303.44	0.26
collagen/riboflavin	299.09	0.55
collagen/riboflavin/gamma-irradiated	299.59	0.79
bovine gelatin/riboflavin	298.96	0.32
bovine gelatin/riboflavin/gamma-irradiated	301.32	0.66
fish gelatin/riboflavin	299.56	0.35
fish gelatin/riboflavin/gamma-irradiated	299.49	0.43

Note: keratin samples were prepared in water; collagen and gelatin samples were prepared in acetic acid 0.18 M.

## Data Availability

Data are contained within the article and Supplementary Materials.

## Supplementary Materials

The following supporting information can be downloaded at: https://www.mdpi.com/article/10.3390/biom13050774/s1: Figure S1: Deconvolution of the amide I region of the IR spectra of (A) collagen and (B) collagen/riboflavin/irradiated; for clarity, band components not belonging to the region of interest are depicted in grey; Figure S2: Deconvolution of the amide I region of the IR spectra of (A) bovine gelatin, (B) bovine gelatin/riboflavin and (C) bovine gelatin/riboflavin/gamma-irradiated; for clarity, band components not belonging to the region of interest are depicted in grey; Figure S3: Deconvolution of the amide I region of the IR spectra of (A) fish gelatin, (B) fish gelatin/riboflavin, (C) fish gelatin/riboflavin/gamma-irradiated; for clarity, band components not belonging to the region of interest are depicted in grey; Figure S4: The CD spectra of collagen/riboflavin (line) and collagen/riboflavin/gamma-irradiated (dotted line) solutions in acetic acid 0.18 M at different temperatures; Figure S5: The μDSC signal for the thermal denaturation of collagen/riboflavin solution in acetic acid 0.18 M, prior to and after gamma irradiation; Table S1: Assignment of the main IR bands of collagen; Table S2: Assignment of the main IR bands of bovine gelatin; Table S3: Assignment of the main IR bands of fish gelatin; Table S4: Secondary structural content of collagen samples; Table S5: Secondary structural content of bovine gelatin samples; Table S6: Secondary structural content of fish gelatin samples; Table S7: Peak temperature and denaturation enthalpy change obtained from μDSC measurements for solutions prepared in acetic acid 15.7 M; Table S8: Parameters of the DMPO and PBN spin adducts formed in protein/riboflavin solutions after exposure to gamma radiation.
