# Supplementary material for: Physico-Chemical Changes Induced by Gamma Irradiation on Some Structural Protein Extracts
Source: Biomolecules. 2023 Apr 29;13(5):774. doi: 10.3390/biom13050774 (PMC10216533; doi:10.3390/biom13050774)
Supplement: Supplementary file 1 [file biomolecules-13-00774-s001.zip › biomolecules-2340893-supplementary.pdf]

Supplementary Material

Physico-Chemical Changes Induced by Gamma Irradiation on Some Structural Protein Extracts

Maria Stanca [1], Carmen Gaidau [1], Traian Zaharescu [2], George-Alin Balan [3], Iulia Matei [3], Aurica Precupas [3], Anca Ruxandra Leonties [3] and Gabriela Ionita [1,3,*]

[1] Leather Research Department, Research and Development National Institute for Textiles and Leather-Division Leather and Footwear Research Institute, 93, Ion Minulescu Street, 031215 Bucharest, Romania
[2] INCDIE ICPE CA, 313 Splaiul Unirii, P.O. Box 149, 030138 Bucharest, Romania
[3] "Ilie Murgulescu" Institute of Physical Chemistry of the Romanian Academy, 202 Splaiul Independentei, 060021 Bucharest, Romania
* Correspondence: gabi2ionita@yahoo.com or ige@icf.ro

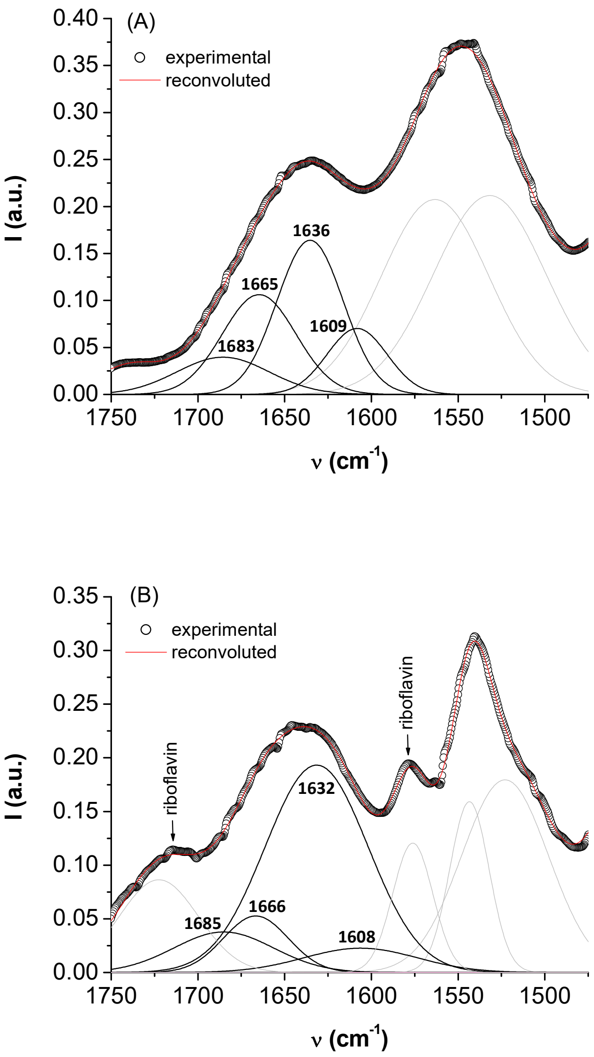

Figure S1. Deconvolution of the amide I region of the IR spectra of (A) collagen, (B) collagen/riboflavin/irradiated; for clarity, band components not belonging to the region of interest are depicted in grey.

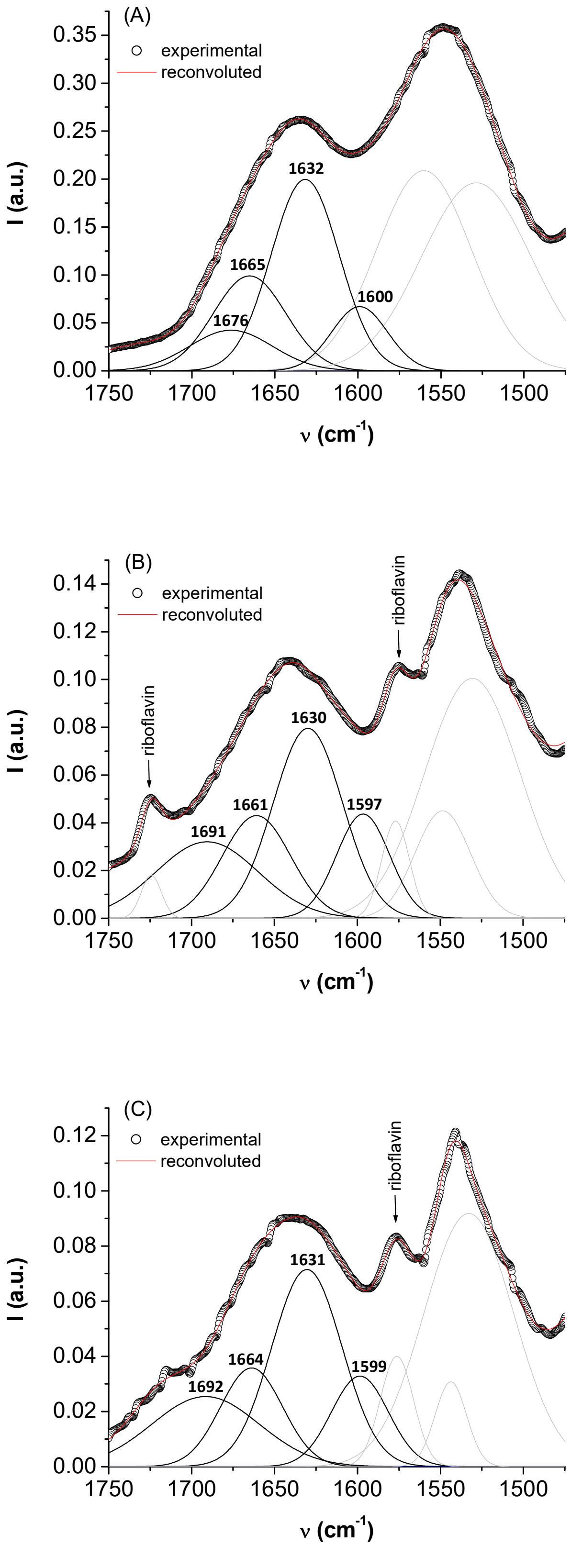

Figure S2. Deconvolution of the amide I region of the IR spectra of (A) bovine gelatin, (B) bovine gelatin/riboflavin, (C) bovine gelatin/riboflavin/gamma irradiated; for clarity, band components not belonging to the region of interest are depicted in grey.

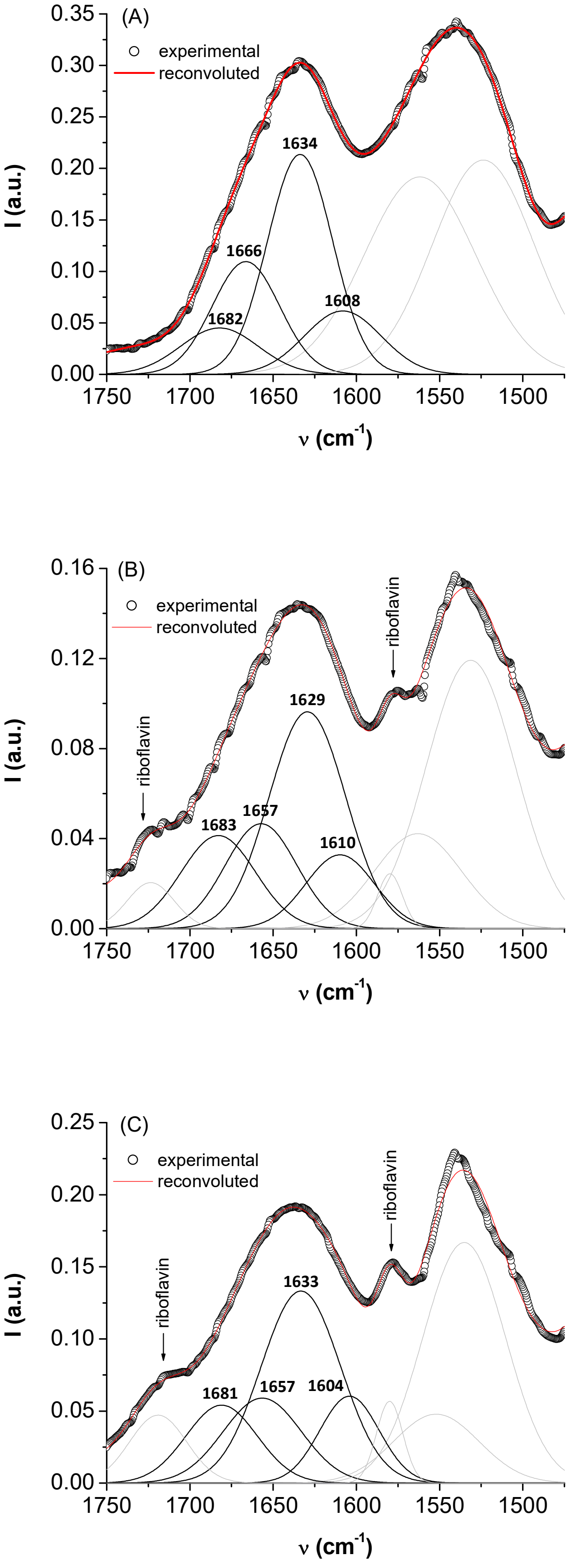

Figure S3. Deconvolution of the amide I region of the IR spectra of (A) fish gelatin, (B) fish gelatin/riboflavin, (C) fish gelatin/riboflavin gamma irradiated; for clarity, band components not belonging to the region of interest are depicted in grey.

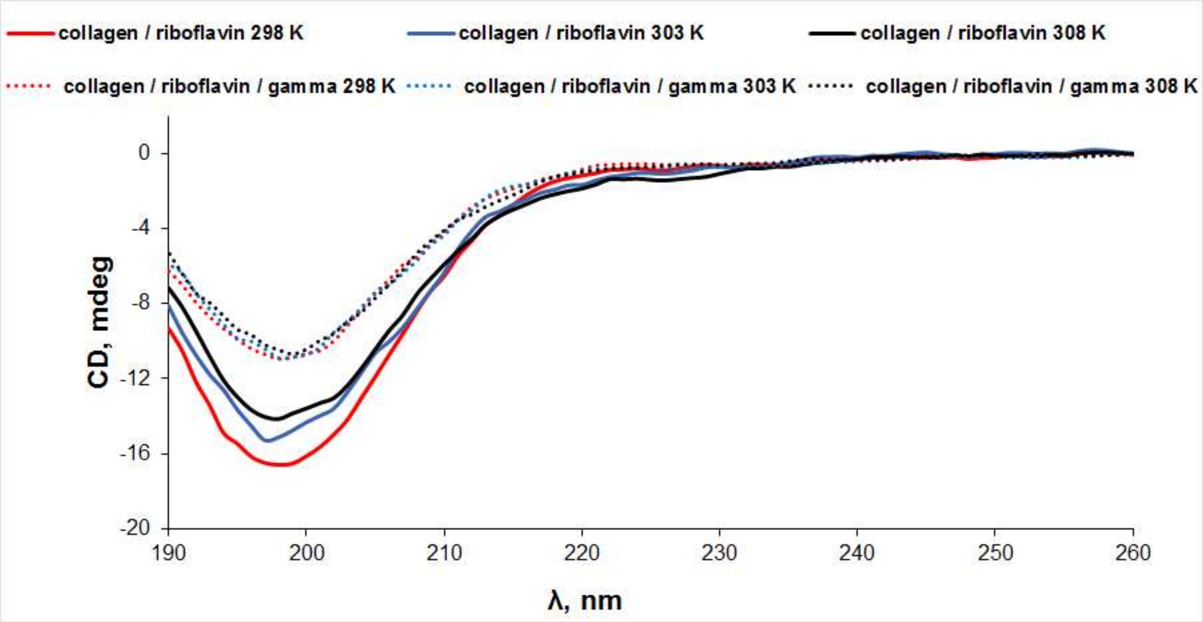

Figure S4. The CD spectra of collagen/riboflavin (line) and collagen/riboflavin/gamma irradiated (dotted line) solutions in acetic acid 0.18 M at different temperatures.

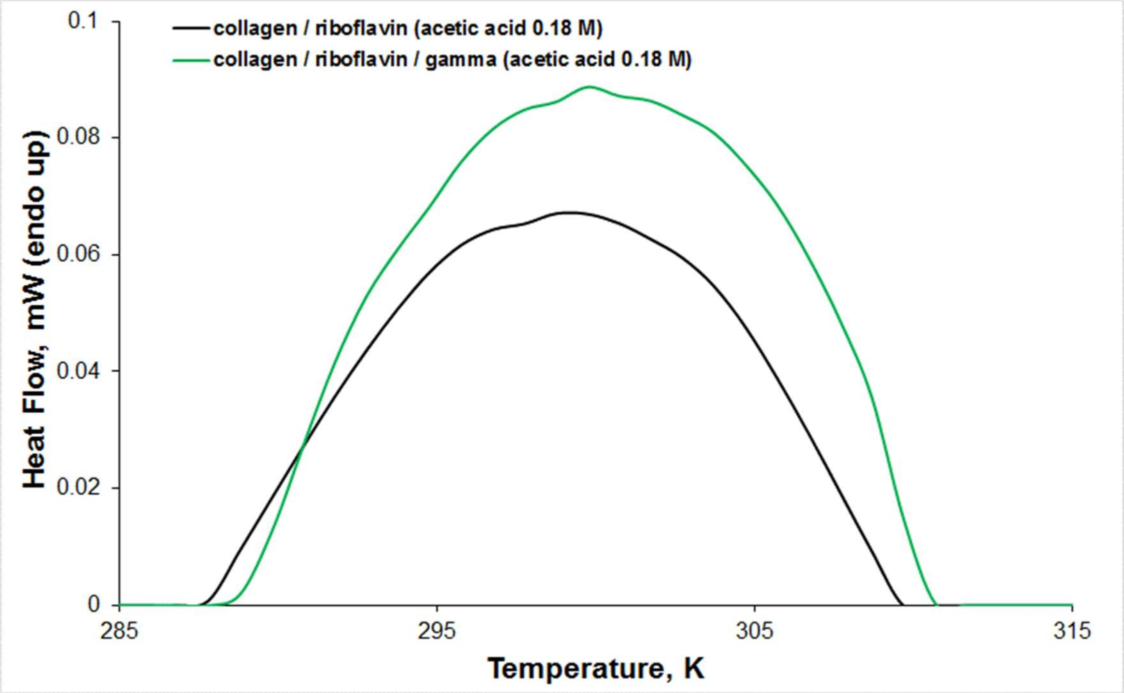

Figure S5. The μDSC signal for the thermal denaturation of collagen/riboflavin solution in acetic acid 0.18 M, prior to and after gamma irradiation.

Table S1. Assignment of the main IR bands of collagen.

Sample	Wavenumber (cm^{-1})	Assignment
collagen	3290, 3076 (w)	O-H, N-H stretching (amide A and B)
	2958 (w), 2923, 2873 (w), 2852	C-H stretching (asymmetric, symmetric)
	1636 (s)	C=O stretching/N-H bending (amide I)
	1547 (s)	C-N stretching/N-H bending (amide II)
	1446, 1408 (s)	C-H bending
	1344, 1242	C-N stretching/N-H bending (amide III)
	1080, 1050, 1020	C-O stretching (glycated collagen)
collagen/riboflavin	3300, 3072 (w)	O-H, N-H stretching (amide A and B)
	2927, 2854	C-H stretching (asymmetric, symmetric)
	1645 (w)	C=O stretching/N-H bending (amide I)
	1538 (s)	C-N stretching/N-H bending (amide II)
	1445, 1407 (s)	C-H bending
	1343, 1240	C-N stretching/N-H bending (amide III)
	1086, 1052, 1021	C-O stretching (glycated collagen)
collagen/riboflavin/gamma	3290, 3069	O-H, N-H stretching (amide A and B)
	2954, 2919 (s), 2972, 2851 (s)	C-H stretching (asymmetric, symmetric)
	1642 (s)	C=O stretching/N-H bending (amide I)
	1540 (s)	C-N stretching/N-H bending (amide II)
	1404 (w)	C-H bending
	1245	C-N stretching/N-H bending (amide III)
	1077, 1060, 1028	C-O stretching (glycated collagen)

Abbreviations: s – strong, w – weak.

Table S2. Assignment of the main IR bands of bovine gelatin.

Sample	Wavenumber (cm^{-1})	Assignment
bovine gelatin	3300, 3071	O-H, N-H stretching (amide A and B)
	2960 (w), 2928, 2875 (w), 2851 (w)	C-H stretching (asymmetric, symmetric)
	1635 (s)	C=O stretching/N-H bending (amide I)
	1549 (s)	C-N stretching/N-H bending (amide II)
	1448 (w), 1409	C-H bending
	1343, 1243	C-N stretching/N-H bending (amide III)
	1080, 1050, 1021	C-O stretching (glycated gelatin)
bovine gelatin/riboflavin	3276, 3069	O-H, N-H stretching (amide A and B)
	2931 (w), 2876 (w)	C-H stretching (asymmetric, symmetric)
	1642 (s)	C=O stretching/N-H bending (amide I)
	1538 (s)	C-N stretching/N-H bending (amide II)
	1454 (w), 1409	C-H bending
	1343, 1251	C-N stretching/N-H bending (amide III)
	1081; 1021	C-O stretching (glycated gelatin)
bovine gelatin/riboflavin/gamma	3300, 3068	O-H, N-H stretching (amide A and B)
	2955 (w), 2918 (s), 2873 (w), 2849 (s)	C-H stretching (asymmetric, symmetric)
	1638 (s)	C=O stretching/N-H bending (amide I)
	1541 (s)	C-N stretching/N-H bending (amide II)
	1448 (w), 1404 (w)	C-H bending
	1350; 1246	C-N stretching/N-H bending (amide III)
	1061, 1031	C-O stretching (glycated gelatin)

Abbreviations: s – strong, w – weak.

Table S3. Assignment of the main IR bands of fish gelatin.

Sample	Wavenumber (cm^{-1})	Assignment
fish gelatin	3275, 3076	O-H, N-H stretching (amide A and B)
	2960 (w), 2933, 2877 (w), 2852 (w)	C-H stretching (asymmetric, symmetric)
	1635 (s)	C=O stretching/N-H bending (amide I)
	1540 (s)	C-N stretching/N-H bending (amide II)
	1446, 1406 (s)	C-H bending
	1338, 1243	C-N stretching/N-H bending (amide III)
	1080, 1050, 1024	C-O stretching (glycated gelatin)
fish gelatin/riboflavin	3279, 3070	O-H, N-H stretching (amide A and B)
	2934, 2877 (w)	C-H stretching (asymmetric, symmetric)
	1634 (s)	C=O stretching/N-H bending (amide I)
	1538 (s)	C-N stretching/N-H bending (amide II)
	1452, 1401	C-H bending
	1343, 1247	C-N stretching/N-H bending (amide III)
	1079, 1061, 1029	C-O stretching (glycated gelatin)
fish gelatin/riboflavin/gamma	3283, 3068	O-H, N-H stretching (amide A and B)
	2955, 2918 (s), 2872, 2851 (s)	C-H stretching (asymmetric, symmetric)
	1638 (s)	C=O stretching/N-H bending (amide I)
	1539 (s)	C-N stretching/N-H bending (amide II)
	1444, 1403	C-H bending
	1347, 1245	C-N stretching/N-H bending (amide III)
	1077, 1057, 1028	C-O stretching (glycated gelatin)

Abbreviations: s – strong, w – weak.

Table S4. Secondary structure content of collagen samples.

Sample	Band Assignment	ν (cm^{-1})	% Area
collagen	β-sheet	1609	15
	random coil	1636	41
	α-helix	1665	30
	β-turn	1683	14
collagen/riboflavin/gamma	β-sheet	1608	8
	random coil	1632	68
	α-helix	1666	12
	β-turn	1685	12

Table S5. Secondary structure content of bovine gelatin samples.

Sample	Band Assignment	ν (cm^{-1})	% Area
bovine gelatin	β-sheet	1600	13
	random coil	1632	48
	α-helix	1665	26
	β-turn	1676	13
bovine gelatin/riboflavin	β-sheet	1597	17
	random coil	1630	40
	α-helix	1661	21
	β-turn	1691	22
bovine gelatin/riboflavin/gamma	β-sheet	1599	16
	random coil	1631	43
	α-helix	1664	19
	β-turn	1692	23

Table S6. Secondary structure content of fish gelatin samples.

Sample	Band Assignment	ν (cm^{-1})	% Area
fish gelatin	β-sheet	1608	16
	random coil	1634	47
	α-helix	1666	25
	β-turn	1682	12
fish gelatin/riboflavin	β-sheet	1610	14
	random coil	1629	46
	α-helix	1657	21
	β-turn	1683	19
fish gelatin/riboflavin/gamma	β-sheet	1604	16
	random coil	1633	47
	α-helix	1657	20
	β-turn	1681	17

Table S7. Peak temperature and denaturation enthalpy change obtained from μDSC measurements for solutions prepared in acetic acid 15.7 M.

Sample	T_{peak} (K)	ΔH (J/g)
keratin	300.75	1.12
keratin/riboflavin/gamma	300.79	0.95
collagen	303.84	2.56
collagen/riboflavin/gamma	298.07	0.21
bovine gelatin	302.64	2.43
bovine gelatin/riboflavin/gamma	301.45	1.19
fish gelatin	303.64	2.14
fish gelatin/riboflavin/gamma	303.14	0.75

Table S8. Parameters of the DMPO and PBN spin adducts formed in protein/riboflavin solutions after exposure to gamma radiation.

	DMPO				PBN			
**Sample**	**1 hour**		**2 hours**		**1 hour**		**2 hours**	
	a_N (G)	a_H (G)	a_N (G)	a_H (G)	a_N (G)	a_H (G)	a_N (G)	a_H (G)
**keratin**	-	-	15.00	14.73	-	-	-	-
**collagen**	14.80	13.97	14.80	14.04	14.70	2.76	14.85	3.15
**bovine gelatin**	14.75	13.86	14.75	13.90	14.61	2.56	14.75	2.95
**fish gelatin**	14.70	13.80	14.75	13.95	14.55	2.50	14.65	2.60
